# Open pneumothorax with extensive thoracic defects sustained in a fall: a case report

**DOI:** 10.1186/s40792-022-01555-x

**Published:** 2022-10-27

**Authors:** Rina Tokuda, Yohei Okada, Futoshi Nagashima, Makoto Kobayashi, Wataru Ishii, Ryoji Iizuka

**Affiliations:** 1 Tajima Emergency and Critical Care Medical Center, Toyooka Public Hospital, 1094 Tobera, Toyooka, Hyogo 668-8501 Japan; 2 Department of Emergency and Critical Care Medicine, Japanese Red Cross Society Kyoto Daini Hospital, 355-5 Haruobicho Kamigyoku, Kyoto, 602-8026 Japan; 3 Department of Preventive Services, School of Public Health, Kyoto University, Yoshida-Konoe-Cho, Sakyo-ku, Kyoto, 606-8501 Japan; 4 Department of Primary Care and Emergency Medicine, Graduate School of Medicine, Kyoto University, Yoshidahon-machi, Sakyo-ku, Kyoto City, Kyoto 606-8501 Japan

**Keywords:** Open pneumothorax, Tracheal intubation, Thoracic drainage, Wound closure, Quick response

## Abstract

**Background:**

Open pneumothorax with chest wall deficit is a rare chest trauma that is serious and can lead to severe respiratory failure; however, it is a potentially lifesaving injury if utilized appropriately.

**Case presentation:**

Herein, we report a case of an open pneumothorax with extensive chest wall deficit due to falling from a height and highlight the importance of appropriate evaluation and intervention. The patient was a Japanese man in his 50 s who fell from the 6th floor to the 3rd floor while working at a height. The left chest wall was punctured due to injury, the thoracic cavity was open as if a left anterolateral thoracotomy had been performed, and the left lung had prolapsed from the thoracic cavity to the outside. In our emergency department, tracheal intubation with a double lumen tube for differential positive pressure ventilation and a right thoracic drain were inserted, and an emergency operation was started immediately. A pulmonary suture for lung injury and closure of the left thorax were performed during the surgery. The defect was closed with the remaining tissue, but the anterior thoracic skin with poor blood flow was necrotic, so debridement was undertaken. After his general condition was improved, pedicled latissimus dorsi myocutaneous flap was implanted. He was discharged home on the 63rd hospital day.

**Conclusions:**

Although open pneumothorax is rare and sometimes presents lurid findings, we highlighted that it is important to quickly assess the life-threatening organ injury, perform positive pressure ventilation by tracheal intubation, thoracic drainage, and wound closure simultaneously respond calmly as a team.

**Supplementary Information:**

The online version contains supplementary material available at 10.1186/s40792-022-01555-x.

## Background

Open pneumothorax is a type of traumatic pneumothorax when air accumulates between the chest wall and the lung due to an open chest wound or physical defect caused by trauma [1]. A larger opening of the chest wall can lead to a greater degree of lung collapse and difficulty of breathing, presenting mediastinal flutter and a frail chest. Furthermore, it can be associated with the injuries of adjacent organs, such as the other lung, bronchi, heart, and diaphragm, requiring emergency surgical intervention [1, 2]. Since the incidence of open pneumothorax is relatively rare, there were few reports about severe cases of open pneumothorax with a large defect in the chest wall, respiratory failure, and mediastinal flutter [3–5]. Herein, we report a case of open pneumothorax with respiratory failure and mediastinal flutter due to extensive thoracic deficit caused by free fall from a height and summarized the key points to treat it successfully.

## Case presentation

A previously healthy 50-year Japanese man fell from the 6th floor to the roof of the 3rd floor while working at a height. A protrusion of the scaffolding extensively injured the left side of his chest wall during the fall, leading to the open pneumothorax. The thorax defect was so extensive that paramedics could not implement the chest seal, and the patient was transferred to our emergency department.

### On arrival findings

**Fig. 1:**
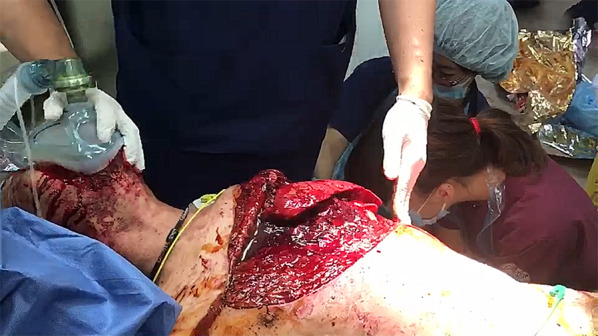
Prolapsed lung-expiratory phase

**Fig. 2:**
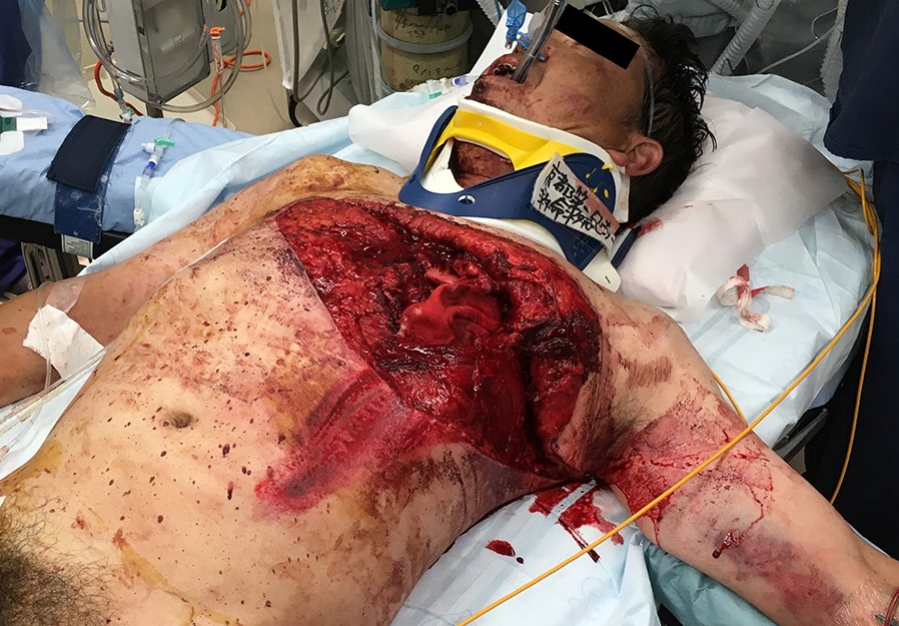
Wound packed with gauze

**Fig. 3:**
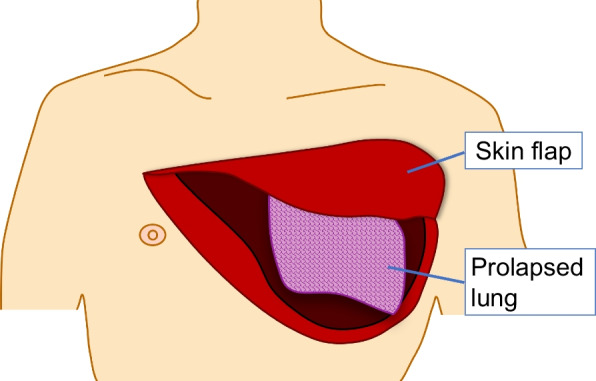
Schematic of the wound

At admission to the emergency department, his airway was open, and his vital signs were respiratory rate: 30 breaths/min, oxygen saturation 97% under 15 L/min mask, blood pressure 161/73 mmHg, pulse 130 bpm, and Glasgow Coma Scale E4V3M6 in restlessness state. There was no apparent subcutaneous emphysema, tracheal displacement, or jugular venous distention in the neck. There was an extensive open wound from the right nipple to the left axilla and dorsal side, as if a left anterolateral thoracotomy had been performed (Figs. 1, 2, 3) (Additional file 1: Movie). A lower lobe of the left lung prolapsed outside the thoracic cavity resulting in difficulty in breathing, mediastinal flutter, and a frail chest. Blood gas analysis under 15 L/min mask was as follows: pH 7.185, pCO_2_ 56.4 mmHg, pO_2_ 194 mmHg, HCO_3_- 20.4 mmol/L, SaO_2_ 98.7%, and Lactate 6.3 mmol/L. Although respiratory sounds were audible on the right side of the chest, a right chest tube was inserted to avoid severe respiratory failure due to bilateral pneumothorax and tension pneumothorax caused by positive pressure ventilation. He was intubated to perform positive pressure ventilation for lung collapse due to left open pneumothorax by the double-lumen tube for isolated lung ventilation in case of emergency surgery to evaluate and repair the associated lung, cardiac diaphragmatic, and other injuries.

**Fig. 4:**
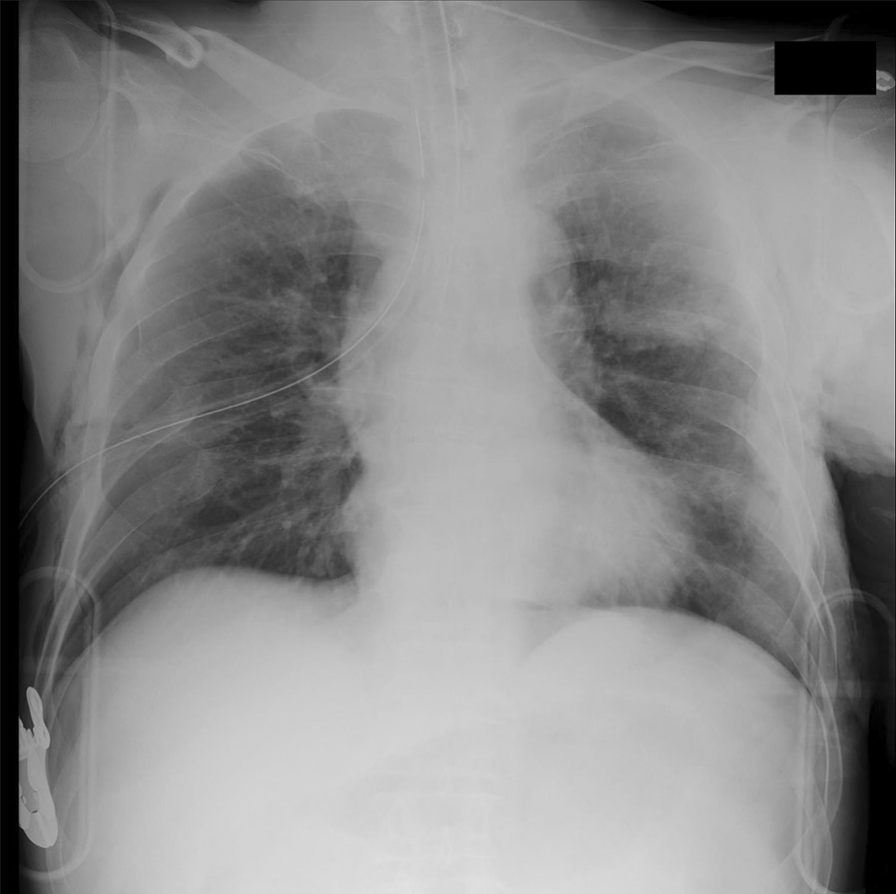
Chest X-ray at primary survey

Focused Assessment with Sonography for Trauma (FAST) was negative, and the chest X-ray showed no extensive pulmonary contusion, massive hemothorax, or multiple rib fractures of the right lung (Fig. 4). The left lung could not be evaluated due to thoracic injury, and no pelvic fractures were observed.

After the intubation and chest tube insertion, we confirmed that airway, respiration, and circulation were stable, and we performed a whole-body computed tomography (CT) scan before surgery. It revealed no apparent extensive pulmonary contusion, massive hemothorax in the right chest, or large vessel injury. However, traumatic subarachnoid hemorrhage, mandibular fracture, dental injury, right clavicle fracture, mediastinal emphysema, left thoracic injury, bilateral pulmonary contusions, left lung laceration, left open pneumothorax, and bilateral multiple rib fractures were observed.

He was moved to the operating room 44 min after arrival at the emergency department to repair the left lung injury and thoracic defect.

### Intraoperative findings

**Fig. 5:**
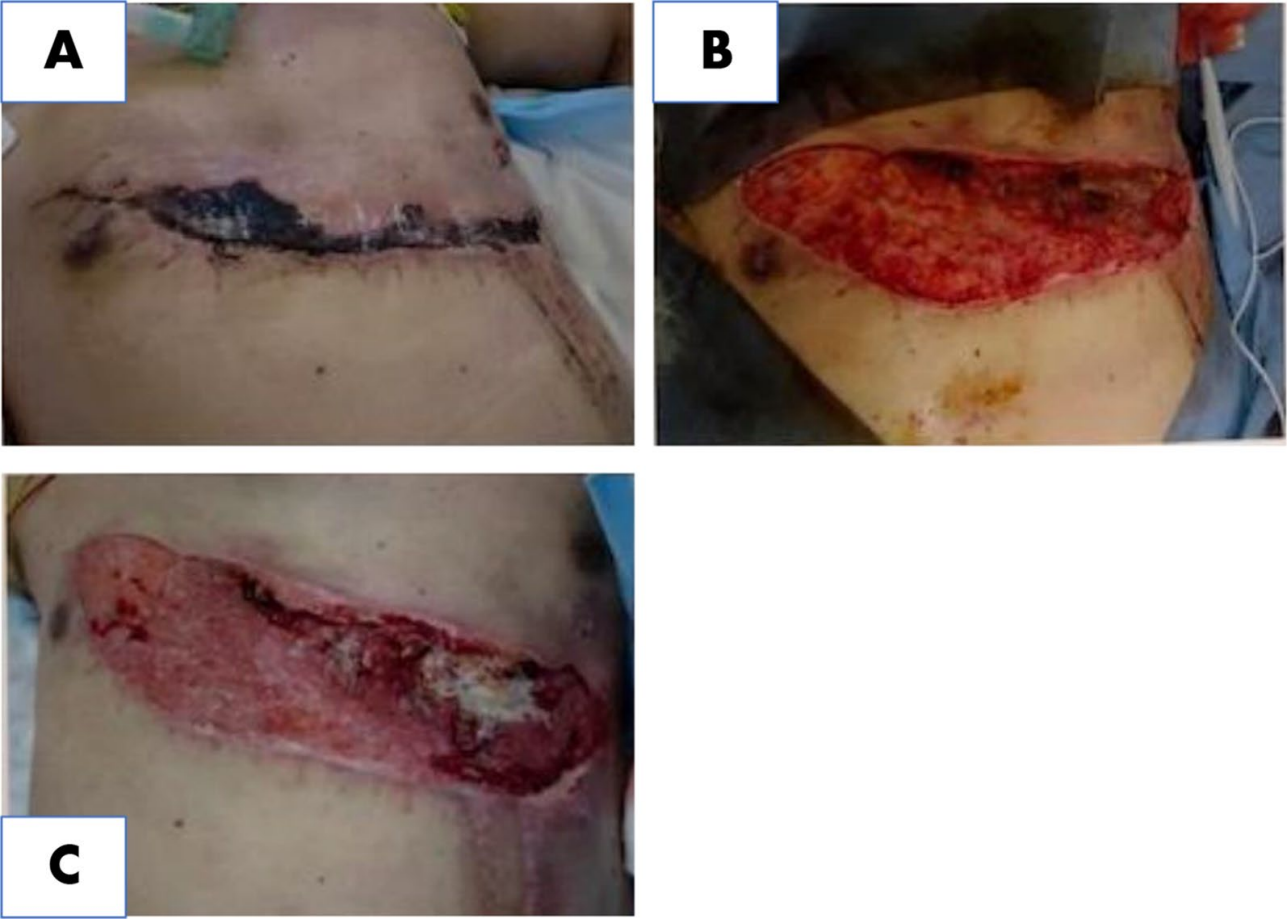
**A** Partial necrosis of the wound, 13 days after initial operation. **B** Wound after debridement, 13 days after initial operation. **C** Defective granulation regenerated, 23 days after initial operation

**Fig. 6:**
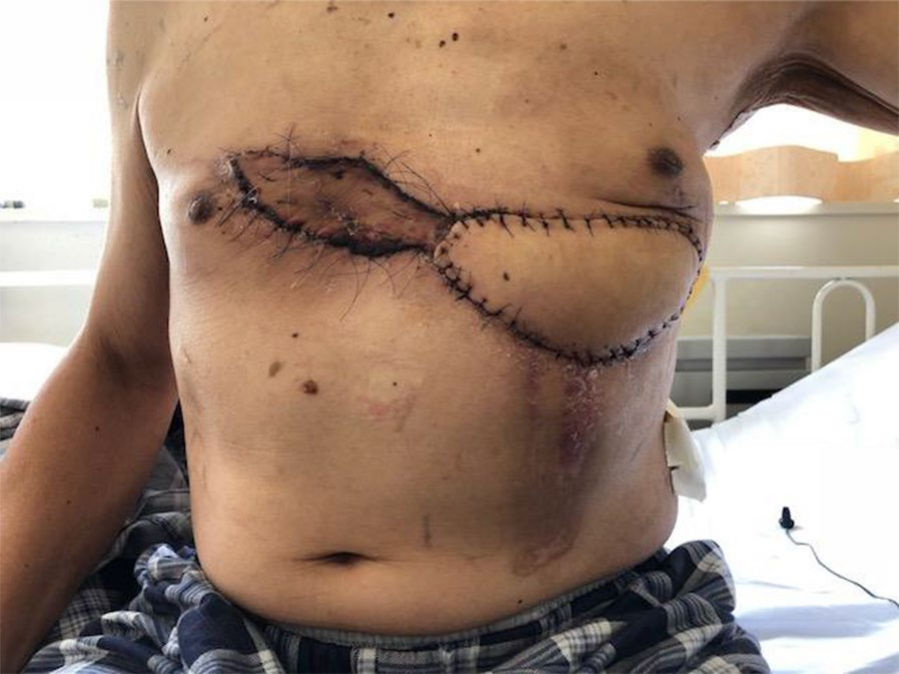
Post-reconstructive chest

**Fig. 7:**
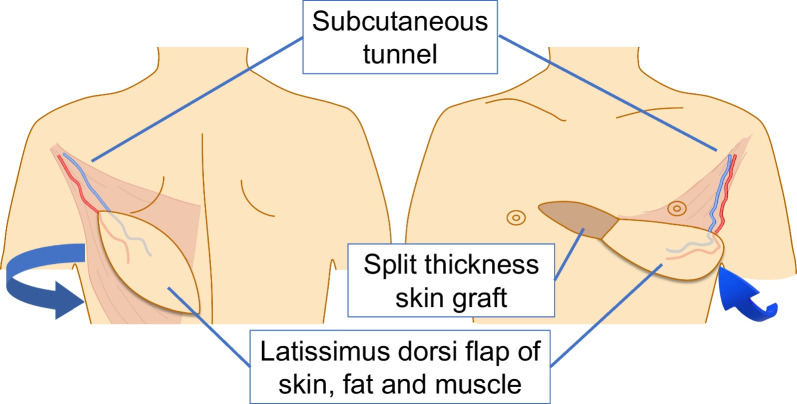
Schematic of thoracic reconstruction with a pedicled latissimus dorsi myocutaneous flap

He was placed in a supine position under general anesthesia with isolated lung ventilation, and anterolateral thoracotomy was performed using a left lateral chest open wound. After the left inferior pulmonary ligament was dissected, we investigated the lung, heart, trachea, diaphragm, and vascular injuries. Six lacerations with air leakage in the left upper lung lobes S4 and S5 were sutured and repaired. No apparent injury was observed on the trachea, bronchi, great vessels, heart, or diaphragm. We flushed the thoracic cavity with normal saline, and after the chest tube was inserted in the left chest, we closed the open wound using soft tissue and muscle. Double-lumen intubation tube changed to single postoperatively. At the same time as the initial surgery, antibiotics were administered to target indigenous skin bacteria and tetanus bacteria. Ventilator-associated pneumonia occurred, because the patient's cough reflex was decreased due to injury of respiratory muscles and sedative medications; however, antibiotic therapy improved it. He was weaned from the ventilator on the 20th hospital day. In addition, debridement was performed due to skin necrosis in part of the closed wound. After he’s general condition improved, a pedicled latissimus dorsi myocutaneous flap was implanted. A 7-cm wide dorsal skin incision area was determined to match the skin defect site on the left thorax. The vastus lateralis muscle was dissected and elevated, and the thoracodorsal artery was traced from the serratus anterior branch to the main trunk, and the vessel was elevated until it reached the thoracic defect, and the pivot point was determined. The lateral thoracic skin was dissected and a vastus lateralis skin valve was passed under the subcutaneous tunnel and placed in the left thoracic wall defect. Excess skin from the dorsal wound closure was excised and a segmental skin graft was placed over the right thoracic defect (Figs. 5, 6, 7). He was discharged home on the 63rd day.

## Discussion

This case reported the case of open pneumothorax with respiratory failure and mediastinal flutter due to extensive chest wall defect caused by fall trauma. Open pneumothorax is widely known as a fatal chest injury [1]. When an open wound of more than two-thirds of the diameter of the bronchus is created in the thorax, it allows the thoracic cavity to interact with the atmosphere. Air enters the thoracic cavity through the open wound due to negative pressure during inhalation and exits through the open wound during expiration [1]. This condition results in inadequate ventilation of the lungs and hypoxemia and requires immediate intervention such as positive pressure ventilation with tracheal intubation to save the patients’ life [1, 6].

The frequency of this injury is relatively rare, and most of them occur on the battlefield and have been reported to occur in only 6% (58 of 992) of penetrating thoracic trauma cases [3]. Open pneumothorax causes are predominantly penetrating injuries and only 1 of 515 cases are blunt chest traumas [4]. Therefore, it is relatively rare to encounter a case of open pneumothorax due to blunt trauma in an urban situation. Therefore, inexperienced physicians and medical teams may be intimidated by the appearance of seriousness, especially if they encounter the open pneumothorax with extensive thoracic defects, prolapsed lung outside the chest cavity, respiratory failure, and mediastinal flutter.

We suggested the three critical points for lifesaving through this case.

First, we highlighted the importance of rapid evaluation and treatment of open pneumothorax. The treatment of open pneumothorax is thoracic drainage and closure of the open wound to the thoracic cavity [1]. At the same time, if respiratory failure due to collapsed lung occurs with mediastinal flutter or frail chest, it should be treated by tracheal intubation and positive pressure ventilation [1]. Especially, since it has been reported that closing the wound of an open pneumothorax can lead to a tension pneumothorax, it is vital to perform these procedures promptly or simultaneously to avoid tension pneumothorax [1, 7]. In this case, we could not accurately assess the right lung condition from the physical examination. Therefore, right thoracic drainage was performed simultaneously to prevent rapid deterioration of respiratory status due to bilateral pneumothorax and to avoid tension pneumothorax associated with positive pressure ventilation. It may be one of the key points in this case.

Second, we also highlighted the importance of rapid evaluation and treatment associated injuries of vital organs such as hemopneumothorax of the contralateral lung, large vessel injury or tracheal/bronchial injury, and massive hemorrhage without getting caught up in the appearance of seriousness. Open pneumothorax has been reported to have a mortality rate of 11.4% (4 out of 35 cases); however, this old data was derived during World War II and could be due to hemorrhage or infection [8]. In addition, a 2013 report from Iraq and Afghanistan showed a mortality rate of 10.5% for all chest trauma, with chest vascular injuries and flail chest being the most common causes [9]. These reports suggest that the cause of death from open pneumothorax is not only respiratory failure caused by the open pneumothorax itself but also organ damage, bleeding, and infection. Therefore, open pneumothorax should be evaluated promptly and lead to the appropriate surgical treatment if they have organ injury and bleeding. In this case, we intubated the patient with an isolated lung ventilation tube, so that we could move quickly to a radical surgical treatment option.

Third, teamwork and a multidisciplinary approach are essential for rapid evaluation and prompt intervention for the open pneumothorax. Obviously, teamwork is essential when dealing with critically ill trauma patients, including appropriately sharing information with the entire team and assigning the roles to perform interventions simultaneously [10]. In the case of open pneumothorax, multiple interventions should be performed simultaneously and rapidly, including tracheal intubation, positive pressure ventilation, chest tube insertion, and wound closure. In addition, prompt surgery is necessary if the other organs are injured, which needs additional surgical teams, anesthesiologists, and other staff in the operation rooms. In point of that, leadership and teamwork is essential to save such severe trauma. Through this case, we believe that these three points are critical issues in treating open pneumothorax.

## Conclusions

This was a case of open pneumothorax with extensive thoracic injury. Although open pneumothorax is rare in occurrence and treatment and sometimes presents with apparent seriousness, it is important to promptly assess the site of injury and damage to other organs, simultaneously perform positive pressure ventilation with tracheal intubation, thoracic drainage, and wound closure, and to respond calmly as a team.

## Supplementary Information

**Additional file 1:** Video of the patient at our emergency department.

## Data Availability

Not applicable.
